# Slow rewarming improved the neurological outcomes of prolonged mild therapeutic hypothermia in patients with severe traumatic brain injury and an evacuated hematoma

**DOI:** 10.1038/s41598-018-30119-z

**Published:** 2018-08-02

**Authors:** Tadashi Kaneko, Motoki Fujita, Susumu Yamashita, Yasutaka Oda, Eiichi Suehiro, Kenji Dohi, Shunji Kasaoka, Yasuhiro Kuroda, Hitoshi Kobata, Tsuyoshi Maekawa

**Affiliations:** 1 Emergency and General Medicine, Kumamoto University Hospital, 1-1-1 Honjo, Chuo-ku, Kumamoto, 860-8556 Japan; 2 Advanced Emergency and Critical Care Center, Yamaguchi University Hospital, 1-1-1 Minamikogushi, Ube, Yamaguchi 755-8505 Japan; 3 Department of Emergency Medicine, Tokuyama Central Hospital, 1-1 Koda-cho, Shunan, Yamaguchi 745-8522 Japan; 4 Department of Emergency, Critical Care and Disaster Medicine, Showa University, School of Medicine, 1-5-8 Hatanodai, Shinagawa-ku, Tokyo 142-8555 Japan; 5 Department of Emergency, Disaster and Critical Care Medicine, Kagawa University Hospital, 1750-1 Ikenobe, Miki-cho, Kita, Kagawa 761-0793 Japan; 6 Osaka Mishima Emergency Critical Care Center, 11-1 Minamiakutagawa-cho, Takatsuki, Osaka, 569-1124 Japan; 7 Yamaguchi Prefectural Grand Medical Center, 77 Osaki, Hofu, Yamaguchi 747-8511 Japan

## Abstract

Mild therapeutic hypothermia (MTH) is expected to improve the neurological outcomes of patients with severe traumatic brain injury (TBI). However, there are no standard protocols for managing the temperature of patients with severe TBI in order to improve their neurological outcomes. We conducted a *post hoc* analysis of the B-HYPO study, a randomized controlled trial of MTH in patients with TBI in Japan. We evaluated the impact of MTH methods on neurological outcomes. Ninety-seven patients who received MTH were included in the present analyses. The neurological outcomes were compared among subgroups of patients divided by cutoff values for the induction, maintenance, and rewarming times of MTH in all patients, in patients with diffuse injury, and in patients with an evacuated hematoma. The proportion of patients with a good neurological outcome was significantly different between patients with an evacuated hematoma divided into subgroups by the cutoff value of rewarming time of 48 h (>48 h vs. ≤ 48 h: 65% vs. 22%; odds ratio: 6.61; 95% confidence interval: 1.13–38.7, *P* = 0.0498). Slow rewarming for >48 h might improve the neurological outcomes of prolonged MTH in patients with TBI and an evacuated hematoma. Further studies are needed to investigate the optimal rewarming protocol in patients with TBI.

## Introduction

Although mild therapeutic hypothermia (MTH) has long been expected to improve the neurological outcomes of patients with severe traumatic brain injury (TBI), two major randomized controlled trials (RCT) did not find any advantages of MTH in this setting^1,2^. However, there are no standard protocols for managing the temperature of patients with severe TBI in order to improve their neurological outcomes. Nevertheless, several *post hoc* analyses of major RCTs suggested that pre-surgical induction of MTH potentially improved the neurological outcomes in patients who had evacuation of a hematoma^3,4^.

To complicate the situation, different MTH protocols are used in patients with other forms of brain damage, and these have yielded different outcomes. For example, MTH (33.5 °C) for 72 h was associated with favorable neurological outcomes in neonates with hypoxic encephalopathy^5^, but MTH (32.5 °C) for 24 h did not have positive results in children with TBI^6^.

The Brain Hypothermia (B-HYPO) study was a Japanese RCT of MTH for severe TBI, in which the temperature management protocol involved a rapid induction phase (≤6 h), a prolonged maintenance phase (≥3 days), and a slow rewarming phase (<1.0 °C/day)^7^. Although the B-HYPO study found no beneficial effect of MTH (32.0–34.0 °C) compared with fever control (34.5–37.0 °C) in neurological outcome, MTH had statistically significant benefits in neurological outcome of patients with an evacuated hematoma^4^. Therefore, we re-analyzed the data from the MTH group to evaluate the impact of the durations of the induction, maintenance, and rewarming phases on the neurological outcomes of patients with TBI.

## Materials and Methods

### Study design and patients

We conducted *post hoc* analyses of patients in the MTH group enrolled in the B-HYPO study. The B-HYPO study was performed between December 2002 and September 2008. It was designed as a multicenter RCT with prospective analyses and blinded assessment of neurological outcomes. The protocol and consent procedures were approved by the Institutional Review Board of each participating hospital (all hospitals are listed at the end of the article as “Collaborating hospitals”), and all experiments were performed in accordance with relevant guidelines and regulations. The study was registered with the University Hospital Medical Information Network (UMIN C000000231 (13/09/2005): https://upload.umin.ac.jp/cgi-open-bin/ctr_e/ctr_view.cgi?recptno=R000000293) in Japan and the National Institutes of Health (Clinical Trials.gov Identifier NCT00134472 (24/08/2005): https://clinicaltrials.gov/ct2/show/NCT00134472?cond=NCT00134472&cntry1=ES%3AJP&rank=1) in the United States of America. The written informed consents were obtained from all inclusion cases. The randomization list was automatically generated by the UMIN computer system to allocate patients in a 2:1 ratio to receive MTH (32–34 °C) or fever control (35.5–37 °C). Males and females were eligible if they were aged 15–69 years, had a Glasgow Coma Scale (GCS)^8^ score of 4–8, and cooling could be started <2 h after the onset of TBI. Patients with any of the following were excluded: good motor response (GCS motor response score 6), systolic blood pressure <90 mmHg after fluid and vasopressor resuscitation, platelet count <50,000 /mm^3^, severe pre-existing medical conditions (e.g., liver, kidney, or heart failure, or severe arrhythmia), acute myocardial infarction, pregnancy, severe alcohol intoxication that prevented assessment of consciousness, penetrating brain injury, epidural hematoma without brain parenchymal injury, or core body temperature <30 °C. Eligible patients were enrolled and their core temperature was lowered to the target temperature as quickly as possible. Computed tomography (CT) of the head was performed and evaluated on admission and after rewarming in all patients.

### Treatments

The core body temperature was measured by a thermister coupled to an internal jugular venous catheter. If the catheter could not be inserted at this site, body temperature was measured at another site that was selected in the following order: pulmonary artery, bladder, rectum, and tympanic membrane. In addition to critical neurological care, an arterial catheter, a pulmonary arterial catheter, and an intracranial pressure (ICP) monitoring probe were inserted to monitor and maintain the patient’s hemodynamic status and ICP at the following levels: mean arterial pressure >80 mmHg, cardiac index >2.5 L/min/m^2^, systemic vascular resistance index (SVRI) 800–1200 dynes/s/cm^5^, ICP < 20 mmHg, and cerebral perfusion pressure >60 mmHg. The partial pressures of arterial oxygen and carbon dioxide were maintained at >100 mmHg and 30–40 mmHg, respectively. If ICP was >20 mmHg, any treatment recommended by the Japanese guidelines^9^ could be administered, including mannitol/glycerol and/or a bolus infusion of barbiturates. Continuous infusion of barbiturates was prohibited due to its severe suppression of cardiac function. Hyperventilation was allowed, but excessive hypocapnia <30 mmHg was prohibited. The ICP could also be reduced by surgical removal of the patient’s cranial bone flap. Anti-convulsants were allowed as deemed necessary by the attending physician.

Cooling blankets, rapid cold fluid infusion (up to 1000 ml of saline, human plasma products, or dextrose-free plasma expanders), and/or cold gastric lavage could be used during the induction phase in both groups. The aim was to achieve the target temperature <6 h after the onset of TBI. The target temperature was to be maintained for ≥72 h, mainly using surface cooling blankets, in both groups. The patient was rewarmed at a rate of <1 °C/day and the core body temperature was maintained at <38 °C for 7 days after the onset of TBI.

Sedatives and analgesics were usually tapered off once the patient had been rewarmed to 36 °C. The muscle relaxant was stopped when the patient had stopped shivering, which usually occurred during the maintenance phase. Muscle relaxants were restarted as deemed necessary by the attending physician.

### Data collection and study outcomes

All data, except the head CT data, were sent to the UMIN center via an internet-based system. The injury severity score (ISS) and head CT classification were also assessed radiologists and neurosurgeons. In this study, the ISS was also calculated as an abbreviated injury score (AIS). The head CT was classified as follows: diffuse injury grade I, all diffuse injuries without CT findings; diffuse injury grade II, high or mixed density lesions with a volume of <25 ml; diffuse injury grade III, high or mixed density lesions with a volume of <25 ml and compressed or absent basal cisterns; diffuse injury grade IV, high or mixed density lesions with a volume of <25 ml and a midline shift of >5 mm; and evacuated/non-evacuated hematoma, high or mixed density lesions with a volume of >25 ml with or without surgical evacuation^10^. Hemodynamic and laboratory data were recorded on Days 0, 1, and 3, as well as 1 day after rewarming (defined as the day on which the core body temperature reached 36 °C). Clinical values were recorded for each day.

The primary outcome was the Glasgow Outcome Scale (GOS)^11^ at 6 months, and was assessed by a neurosurgeon, neurologist or emergency physician unaware of the patient’s treatment method. One month and 6 months after discharge from the original hospital, the patients were followed-up by telephone. The neurological outcomes were classified as good (moderate disability or good recovery) or poor (severe disability, persistent vegetative state, or death).

### Statistical analyses

In the present study, we compared the proportion of patients with good neurological outcomes between subgroups of patients divided by cutoff values for the induction phase (time taken to reach 34.0 °C ≤6 h), maintenance phase (duration of hypothermia ≥72 h), and rewarming phase (time taken to rewarm to 36.0 °C ≥48 h) in all patients, in patients with diffuse injury, and in patients with an evacuated hematoma. These cutoff values of induction (≤6 h), maintenance (≥72 h), and rewarm (≥48 h), were considered as ideal target of therapeutic hypothermia for severe TBI by our study group and protocol.

Continuous variables were statistically analyzed using the Mann–Whitney *U* test. Categorical variables were statistically analyzed using Fisher’s exact probability test. Multivariate analysis was performed by logistic regression analysis. The threshold of significance was set at *P* < 0.05. Statistical analyses were performed using SPSS software version 23.0 (SPSS Inc., Chicago, IL, USA).

### Collaborating hospitals

Showa University Hospital, Tokyo; Yamaguchi University Hospital, Yamaguchi; Japanese Red Cross Musashino Hospital, Tokyo; Sapporo University Hospital, Hokkaido; Kagawa University Hospital, Kagawa; Osaka Mishima Emergency Critical Care Center, Osaka; Gifu University Hospital, Gifu; Nippon Medical School Hospital, Tokyo; Ehime University Hospital, Ehime; Fujita Health University Hospital, Aichi; Chiba Emergency Medical Center, Chiba; St. Marianna University Hospital, Kanagawa; Saiseikai Utsunomiya Hospital, Tochigi; Kitasato University Hospital, Kanagawa; Kawasaki Medical School Hospital, Okayama; Nara Medical University Hospital, Nara; Chiba University Hospital, Chiba; Nagoya Medical Center, Aichi; The Hospital of Hyogo College of Medicine, Hyogo; National Tokyo Medical Center, Tokyo; Nihon University Hospital, Tokyo; Nippon Medical School Tama Nagayama Hospital, Tokyo; Tokyo Medical University Hachioji Medical Center, Tokyo; Tokyo Medical University Hospital, Tokyo; Aizawa Hospital, Nagano; Shinshu University Hospital, Nagano; Shiga University Hospital, Shiga; Tokushima University Hospital, Tokushima; Toho University Omori Medical Center, Tokyo; Teikyo University Hospital, Tokyo; Saitama Medical Center, Saitama; Osaka National Hospital, Osaka; Kansai Medical University Takii Hospital, Osaka; Ohota Nishinouchi Hospital, Fukushima; Oita University Hospital, Oita; and Iwate Medical University Hospital, Iwate. All collaborating hospitals are in Japan.

## Results

Table 1 shows the characteristics on admission of patients who received MTH, for patients with an evacuated hematoma and for those with diffuse injury. Age, proportion of males, vital signs (blood pressure, heart rate, unreactive pupil/pupils), and GCS score were significantly different between the two groups of patients. However, the trauma severity scores (ISS and AIS for head and other organs) and outcomes (neurological outcomes and mortality rate at 6 months) were not significantly different between the two groups.

**Table 1:** Characteristics of patients with severe TBI on admission, for the evacuated hematoma and diffuse brain injury groups.

Variable	Evacuated hematoma (*N* = 43)	Diffuse injury (*N* = 54)	*P* value
Age (years)	54 (23–63)	27 (20–47)	**0**.**002**
Male	25 (58%)	43 (80%)	**0**.**027**
SBP (mmHg)	160 (123–187)	132 (110–153)	**0**.**001**
DBP (mmHg)	90 (70–104)	79 (65–90)	**0**.**025**
Heart rate (beats/min)	79 (70–94)	100 (77–118)	**0**.**007**
Cardiac index on Day 1 (l/min/m^2^)	2.9 (2.4–3.6)	3.0 (1.5–3.5)	0.617
GCS	5 (4–7)	6 (5–7)	**0**.**021**
GCS score 4–5	24 (56%)	16 (30%)	**0**.**013**
GCS score 6–8	19 (44%)	38 (70%)	
Unreactive pupil(s)	27 (63%)	19 (35%)	**0**.**008**
Head CT grade on admission			
Diffuse injury grade I	0	1 (2%)	
Diffuse injury grade II	0	30 (56%)	
Diffuse injury grade III	0	15 (28%)	
Diffuse injury grade IV	0	3 (6%)	
Evacuated hematoma	43 (100%)	0	
Non-evacuated hematoma	0	5 (9%)	
Surgical treatment of TBI	43 (100%)	18 (33%)	**<0**.**001**
ISS	25 (17–33)	27 (18–35)	0.221
AIS score for head	4 (4–5)	4 (4–5)	0.298
AIS score ≥4 for other organs	4 (9%)	6 (11%)	1
Good neurological outcome (GR or MD rating on GOS)	19/42 (45%)	25/51 (49%)	0.835
Mortality rate	15/42 (36%)	17/51 (33%)	0.83

Values are presented as the median (range) or *n* (%) of patients.

*SBP* systolic blood pressure, *DBP* diastolic blood pressure, *GCS* Glasgow Coma Scale, *CT* computed tomography, *TBI* traumatic brain injury, *ISS* injury severity score, *AIS* abbreviated injury score; *GR* good recovery, *MD* moderate disability, *GOS* Glasgow Outcome Scale.

Tables 2–4 compare the proportions of patients with good neurological outcomes by cutoff values for the induction phase (time taken to reach 34.0 °C ≤6 h), maintenance phase (duration of hypothermia ≥ 72 h), and rewarming phase (time taken to rewarm to 36.0 °C ≥48 h) in all patients (Table 2), patients with diffuse injury (Table 3) and patients with an evacuated hematoma (Table 4). As shown in Table 4, the proportion of patients with an evacuated hematoma who had a good neurological outcome after MTH was significantly greater in patients with a rewarming time of ≥48 h (*P* = 0.0498). This suggests that slow rewarming (i.e., ≥48 h) may confer better neurological outcomes than quicker rewarming.

**Table 2:** Proportions of patients with good neurological outcomes according to cutoff values of the induction, maintenance, and rewarming phases: all patients who received therapeutic hypothermia.

Subgroup of patients	*N*	Good neurological outcome (GR or MD rating on GOS)*	OR (95% CI)	*P* value
Duration of induction phase ≤6 h	87		0.85 (0.35–2.07)	0.822
≤6 h	30	13 (43%)		
>6 h	57	27 (47%)		
Duration of hypothermia phase ≥ 72 h	77		0.92 (0.36–2.40)	1
≥72 h	52	26 (50%)		
<72 h	25	13 (52%)		
Duration of rewarming phase ≥48 h	75		1.89 (0.63–5.65)	0.282
≥48 h	58	33 (57%)		
<48 h	17	7 (41%)		

*Values are presented as the n (%) of patients.

*GR* good recovery, *MD* moderate disability, *GOS* Glasgow Outcome Scale, *OR* odds ratio, *CI* confidence interval.

**Table 3:** Proportions of patients with good neurological outcomes according to cutoff values of the induction, maintenance, and rewarming phases: patients with diffuse brain injury.

Subgroup of patients	*N*	Good neurological outcome (GR or MD rating on GOS)*	OR (95% CI)	*P* value
Duration of induction phase ≤6 h	45		0.33 (0.09–1.28)	0.121
≤6 h	14	4 (29%)		
>6 h	31	17 (55%)		
Duration of hypothermia phase ≥72 h	39		1.75 (0.44–6.93)	0.501
≥72 h	27	15 (56%)		
<72 h	12	5 (42%)		
Duration of rewarming phase ≥48 h	40		0.60 (0.12–2.94)	0.698
≥48 h	32	16 (50%)		
<48 h	8	5 (62%)		

*Values are presented as the n (%) of patients.

*GR* good recovery, *MD* moderate disability, *GOS* Glasgow Outcome Scale, *OR* odds ratio, *CI* confidence interval.

**Table 4:** Proportions of patients with good neurological outcomes according to cutoff values of the induction, maintenance, and rewarming phases: patients with evacuated hematoma.

Subgroup of patients	*N*	Good neurological outcome (GR or MD rating on GOS)*	OR (95% CI)	*P* value
Duration of induction phase ≤6 h	42		2.06 (0.58–7.29)	0.344
≤6 h	16	9 (56%)		
>6 h	26	10 (38%)		
Duration of hypothermia phase ≥72 h	38		0.49 (0.13–1.93)	0.495
≥72 h	25	11 (44%)		
<72 h	13	8 (62%)		
Duration of rewarming phase ≥48 h	35		6.61 (1.13–38.7)	**0**.**0498**
≥48 h	26	17 (65%)		
<48 h	9	2 (22%)		

*Values are presented as the n (%) of patients.

*GR* good recovery, *MD* moderate disability, *GOS* Glasgow Outcome Scale, *OR* odds ratio, *CI* confidence interval.

Table 5 compares the characteristics of patients with an evacuated hematoma divided by rewarming time (<48 h vs. ≥48 h). The proportion of patients with a good neurological outcome (*P* < 0.0498) and the mortality rate (*P* = 0.015) were significantly different between these two groups of patients. There were also some differences, albeit not significant, between the two groups, with age, GCS score on admission, duration of the hypothermia maintenance phase, and initial ICP being younger, more severe, longer, and lower, respectively, in the ≥48 h rewarming group than in the <48 h rewarming group.

**Table 5:** Characteristics of patients with severe traumatic brain injury and an evacuated hematoma according to the duration of the rewarming phase.

Variables	Duration of rewarming phase		*P* value
	≥48 h (*N* = 27)	<48 h (*N* = 9)	
Age (years)	32 (20–59)	59 (52–69)	0.052
Male	14 (52%)	7 (78%)	0.252
SBP (mmHg)	160 (117–187)	144 (113–175)	0.693
DBP (mmHg)	89 (62–106)	90 (69–95)	0.697
Heart rate (beats/min)	80 (72–94)	70 (58–96)	0.16
Cardiac index on Day 1 (l/min/m^2^)	3.7 (3.5–4.8)	3.4 (2.9–5.5)	0.594
GCS	5 (4–7)	6 (4–7)	0.472
GCS score 4–5	17 (63%)	3 (33%)	0.146
GCS score 6–8	10 (37%)	6 (67%)	
Unreactive pupil(s)	18 (67%)	5 (56%)	0.693
Surgical treatment of TBI	27 (100%)	9 (100%)	
ISS	25 (17–34)	25 (17–34)	0.943
AIS score for head	4 (4–5)	4 (4–5)	1
AIS score ≥4 for other organs	3 (11%)	0 (0%)	0.558
Duration of the induction phase (h)	8.5 (4.9–18.5)	8.3 (4.7–14.6)	0.886
Duration of the maintenance phase (h)	76 (68.5–85)	68 (36–79.8)	0.086
Initial ICP (mmHg)	14 (7–40)	66.5 (20.3–91.8)	0.062
ICP during the maintenance phase (mmHg)	15 (12–20)	13 (3–57)	0.739
ICP before rewarming (mmHg)	15 (12–20)	16 (10–83)	0.478
ICP after rewarming (mmHg)	16.5 (11–19)	12 (8.8–40.3)	0.595
Good neurological outcome (GR or MD rating on GOS)	17/26 (65%)	2/9 (22%)	**0**.**0498**
Mortality rate	3/26 (12%)	5/9 (56%)	**0**.**015**

Values are presented as the median (range) or *n* (%) of patients.

*SBP* systolic blood pressure, *DBP* diastolic blood pressure, *GCS* Glasgow Coma Scale, *TBI* traumatic brain injury, *ISS* injury severity score, *AIS* abbreviated injury score; *ICP* intracranial pressure, *GR* good recovery, *MD* moderate disability, *GOS* Glasgow Outcome Scale.

Table 6 shows the results of multivariate analysis of age, duration of the maintenance phase, initial ICP, and good neurological outcome with an evacuated hematoma divided by rewarming time (<48 h vs. ≥48 h). There was no significant difference between the groups.

**Table 6:** Multivariate analysis of patients with severe traumatic brain injury and an evacuated hematoma according to the duration of the rewarming phase.

Variables	Duration of rewarming phase		*P* value	OR (95% CI)
	≥48 h (*N* = 27)	<48 h (*N* = 9)		
Age (years)	32 (20–59)	59 (52–69)	0.194	0.92 (0.8–1.05)
Duration of the maintenance phase (h)	76 (68.5–85)	68 (36–79.8)	0.378	1 (0.998–1.004)
Initial ICP (mmHg)	14 (7–40)	66.5 (20.3–91.8)	0.098	0.95 (0.88–1.01)
Good neurological outcome (GR or MD rating on GOS)	17/26 (65%)	2/9 (22%)	0.422	6.64 (0.07–670.07)

Values are presented as the median (range) or *n* (%) of patients.

*ICP* intracranial pressure, *GR* good recovery, *MD* moderate disability, *GOS* Glasgow Outcome Scale, *OR* odds ratio, *CI* confidence interval.

## Discussion

The present *post hoc* analyses were performed under our desire to improve the temperature management of MTH, because the B-HYPO study involved a long duration of hypothermia (≥72 h; median 75.5 h, range 68.9–84.0 h) and a long rewarming time (mean 76.0 h, range 51.5–113.5 h) owing to the slow rewarming rate of ≤1.0 °C/day in a well-controlled study in terms of age, GCS, hemodynamic control, and inter-/intra-center randomization. We previously reported that patients with an evacuated hematoma experienced better neurological outcomes after MTH than patients with diffuse brain injury^4^. Therefore, we re-analyzed the results of the MTH group in patients with an evacuated hematoma or diffuse injury in the B-HYPO study.

The benefits of slow rewarming from MTH were well documented in animal studies^12,13^, but there was no clinical evidence, except for a systematic review and a guideline to clearly support this approach in humans^14,15^. Usually, the clinical protocols for MTH have focused on the rate of cooling and the duration of MTH, rather than the duration or rate of rewarming. Therefore, we re-analyzed the MTH group of the B-HYPO study to examine the relative impact of each phase (i.e. induction, maintenance, and rewarming phases) on neurological outcomes using clinically appropriate cutoff values for each phase. We found that a rewarming phase of ≥48 h (mean 96 h, range 72–164 h) was associated with a significantly greater rate of good neurological outcomes and significantly lower mortality rate compared with a rewarming period of <48 h (mean 35 h, range 9–43.5 h) in patients with an evacuated hematoma (Table 5). Although the patient characteristics were not significantly different between the two groups, the better outcomes with longer rewarming might be due to small, non-significant differences, particularly the younger age, longer MTH maintenance period, and lower initial ICP in patients with a rewarming time of ≥48 h, although the GCS on admission was more severe in this group than in patients with a rewarming time of <48 h (Table 5). However, the multivariate analysis of these factors (age, duration of the maintenance phase, initial ICP, and good neurological outcome) did not show significance between the group, therefore there might be no evident confounder (Table 6). Young age itself is an independent predictor of a good neurological outcome in patients with TBI, as supported by the present results, and possibly allowed the attending physician to prolong the maintenance and rewarming periods in the present study.

In the present study, we found no significant effects of MTH in terms of the induction time (≤6 h) and duration of hypothermia (≥72 h). The induction times analyzed here are similar to those used in two earlier RCTs (8.4 h and 4.4 h)^1,2^, although the duration of hypothermia differed from that used by Jiang *et al*.^16^. That study revealed that prolonged hypothermia (5 days) was associated with better neurological outcomes, and the authors hypothesized that hypothermia suppressed rebound intracranial hypertension during prolonged hypothermia and rewarming^16^. Although a reduction of ICP by MTH did not improve the neurological outcomes of patients with TBI^16,17^, animal studies revealed that MTH suppressed TBI-related brain damage in both diffuse injury and post hematoma evacuation^12,18^. Nevertheless, recent clinical studies reported that the advantage of MTH on neurological outcomes was only apparent in patients with severe brain injury and an evacuated hematoma^2–4^. These results imply that the neurological outcomes after MTH are dependent on the type of brain damage.

In the present study, ICP was controlled or suppressed during MTH, as in prior studioes^18–20^. The mean (range) initial ICP were 14 mmHg (7–40 mmHg) and 67 mmHg (20–92 mmHg) with rewarming times of ≥48 h and <48 h, respectively (Table 5). In general, the neurological outcomes are extremely poor in patients with TBI whose ICP exceeds 35 mmHg^21^. This finding is especially relevant to patients with a rewarming time of <48 h. The ICP was not significantly different between the two groups of patients in this study, and was to be maintained at <20 mmHg during and after MTH. Nevertheless, the initial ICP was ≥35 mmHg in 3 patients at baseline (i.e., initial ICP), in 3 patients during the maintenance phase, in 3 patients before rewarming, and in 2 patients after rewarming, in the sub-group of patients with a rewarming time of <48 h. These results are similar to those of earlier studies^18–20^. We hypothesize that ischemia/reperfusion of the injured brain tissue might cause recurrent ICP elevation/reduction during the rewarming phase. In the present study, blood flow to the injured brain regions was adequate based on the cardiac index (Table 1), especially in the slow rewarming group (Table 5). Some animal studies have suggested that rapid rewarming aggravated TBI by causing an imbalance between cerebral blood flow and metabolism during the rewarming phase, and induced microcirculatory disorders in the brain via cerebrovascular reactions^22,23^. In the present study, although the ICP and cardiac index were well controlled (Table 5), the rewarming time had an impact on the neurological outcomes, and slow rewarming might help to prevent unwanted increases in ICP and stabilize the systemic hemodynamic variables (Table 5).

This study had some limitations. First, we conducted retrospective, *post hoc* analyses of a previous RCT and the sample size was not adequately powered for the present analyses, which weakens the strength of our findings. Second, the cutoff values were selected to be clinically relevant, and were not determined using statistical methods. Third, the B-HYPO RCT involved a flexible protocol, although the minimum duration of MTH was 72 h and the maximum rewarming rate was 1.0 °C/day. Therefore, some bias may exist in terms of differences in the MTH protocols used in different patients, although the background characteristics were not significantly different between the two patient groups (Table 5).

## Conclusions

In conclusion, the results of the present study suggest that slow rewarming from MTH (i.e., ≥48 h) might be associated with better neurological outcomes than quicker rewarming (i.e., <48 h) in patients with TBI and an evacuated hematoma. Our results also suggest that prolonged rewarming of ≥ 48 h together with frequent measurement of ICP might be an appropriate strategy for preventing rebound intracranial hypertension in patients with TBI and an evacuated hematoma.

## References

[1] Clifton GL (2001). Lack of effect of induction of hypothermia after acute brain injury. N Engl J Med..

[2] Clifton GL (2011). Very early hypothermia induction in patients with severe brain injury (the National Acute Brain Injury Study: Hypothermia II): a randomized trial. Lancet Neurol..

[3] Clifton GL (2012). Early induction of hypothermia for evacuated intracranial hematomas: a post hoc analysis of two clinical trials. J Neurosurg..

[4] Suehiro E (2015). Diverse effects of hypothermia therapy in patients with severe traumatic brain injury based on the computed tomography classification of the traumatic coma data bank. J Neurotrauma..

[5] Shankaran S (2005). Whole-body hypothermia for neonates with hypoxic-ischemic encephalopathy. N Engl J Med..

[6] Hutchison JS (2008). Hypothermia therapy after traumatic brain injury in children. N Engl J Med..

[7] Maekawa T (2015). Prolonged mild therapeutic hypothermia versus fever control with tight hemodynamic monitoring and slow rewarming in patients with severe traumatic brain injury: a randomized controlled trial. J Neurotrauma..

[8] Jennett B, Teasdale GM (1973). Assessment of coma and impaired consciousness: a practical scale. Lancet..

[9] The Japan Society of Neurotraumatology. Guidelines for the Management of Severe Head Injury (Igaku-Shoin, Tokyo, 2001).

[10] Marshall LF, Gautille T, Klauber MR (1991). The outcome of severe closed head injury. J Neurosurg..

[11] Jennett B, Snoek J, Bond MR, Brooks N (1981). Disability after severe head injury: observations on the use of the Glasgow outcome scale. J Neurol Neurosurg Psychiat..

[12] Povlishock JT, Wei EP (2009). Posthypothermic rewarming considerations following traumatic brain injury. J Neurotrauma..

[13] Enomoto S, Hindman BJ, Dexter F, Smith T, Gutkomp J (1996). Rapid rewarming causes an increase in the cerebral metabolic rate for oxygen that is temporarily unmatched by cerebral blood flow: a study during cardiopulmonary bypass in rabbits. Anesthesiology..

[14] McIntyre LA, Fergusson DA, Hebert PC, Moher D, Hutchison JS (2003). Prolonged therapeutic hypothermia after traumatic brain injury in adults: a systematic review. JAMA..

[15] Brain Trauma Foundation. American Association of Neurological Surgeons. Congress of Neurological Surgeons. Joint Section on Neurotrauma and Critical Care. AANS/CNS. Guidelines for the management of severe traumatic brain injury III. Prophylactic hypothermia. *J Neurotrauma*. **24**, S21–25 (2007).

[16] Jiang JY (2006). Effect of long-term mild hypothermia or short-term mild hypothermia on outcome of patients with severe traumatic brain injury. J Cereb Blood Flow Metab..

[17] Andrews PJ (2015). Hypothermia for intracranial hypertension after traumatic brain injury. N Engl J Med..

[18] Sandestig A, Rommer B, Grände PO (2014). Therapeutic hypothermia in children and adults with severe traumatic brain injury. Ther Hypothermia Temp Manag..

[19] Schreckinger M, Marion DW (2009). Contemporary management of traumatic intracranial hypertension: is there a role for therapeutic hypothermia?. Neurocrit Care..

[20] Flynn, L. M., Rhodes, J. & Andrews, P. J. Therapeutic hypothermia reduced intracranial pressure and partial brain oxygen tension in patients with severe traumatic brain injury: preliminary data from the eurothem3235 trial. *Ther Hypothermia Temp Manag*. **5**, 143–151 (2015). 10.1089/ther.2015.0002 PMC4575517 26060880

[21] Chambers IR, Treadwell L, Mendelow AD (2001). Determination of threshold levels of cerebral perfusion pressure and intracranial pressure in severe head injury by using receiver-operating characteristic curves: an observational study in 291 patients. J Neurosurg..

[22] Suehiro E, Ueda Y, Wei EP, Kontos HA, Povlishock JT (2003). Posttraumatic hypothermia followed by slow rewarming protects the cerebral microcirculation. J Neurotrauma..

[23] Nakamura T (1999). Influence of rewarming conditions after hypothermia in gerbils with transient forebrain ischemia. J Neurosurg..

